# 78595 Assessing the influence of comorbidities in patients undergoing sternal reconstruction following cardiac surgery: a single institution's 15 year review

**DOI:** 10.1017/cts.2021.717

**Published:** 2021-03-30

**Authors:** Edgar Soto, Pallavi A. Kumbla, Ryan Restrepo, Thomas K. Delay, Shadi K Awad, Sherry Collawn, Jorge de la Torre, Brad Denney, Jobe R Fix, John H Grant, Ali Kilic, Timothy W King, Prasanth Patcha, James Davies, Luis O Vasconez, Rene P. Myers

## Abstract

ABSTRACT IMPACT: Current practice guidelines offer a variety of treatment options for sternal reconstruction but complications and infections remain a serious surgical problem. This work seeks to provide a comprehensive picture of the com-morbidities and reconstructive methods that lead to success and improve patient outcomes. OBJECTIVES/GOALS: Patients that undergo cardiac surgery via the median sternotomy approach are at risk of wound complications that require repair. We seek to evaluate how outcomes of sternal reconstruction are influenced by patient comorbidities, flap usage and internal mammary artery grafts and methods of sternal closure. METHODS/STUDY POPULATION: We identified patients between 2005 and 2020 who underwent sternotomy followed by debridement and flap coverage at our institution. Comorbidities, method of reconstruction, demographic data, surgical history, and other factors pertaining to mortality and morbidity were collected. The data will then be analyzed to identify population characteristics using logistic regression variables to determine univariate and adjusted multivariable measures of association with mortality. We present the pre-liminary data analyzed using chi-square and one-way anova in R. RESULTS/ANTICIPATED RESULTS: In this study we present a preliminary characterization of one institution’s sternal reconstruction patient outcomes with a variety of reconstruction methods including pectoralis advancement flaps, omental flaps and latissumus dorsi flaps. Notable preoperative comorbidities include 50% of patients > age 60, 18% with diabetes mellitus, 18 % with diagnosed hypertension, 18% with COPD, and 9% with a smoking history DISCUSSION/SIGNIFICANCE OF FINDINGS: In an evolving cardiothoracic landscape, clinical characteristics of patients being treated for sternal reconstructive surgery present a moving target. Understanding current risk factors, preoperative management and timing for aggressive surgical treatment offers an opportunity to update treatment protocol and maximize successful outcomes.

